# Effect of melatonin on steroidogenesis-related enzymes expression and testosterone synthesis following CoCl_2_-induced hypoxia in TM3 Leydig cells

**DOI:** 10.22038/IJBMS.2023.69570.15152

**Published:** 2023

**Authors:** Shokooh Karimi, Cyrus Jalili, Kamran Mansouri, Fariborz Bahremand, Mohammad Reza Gholami

**Affiliations:** 1 Department of Anatomical Sciences, Kermanshah University of Medical Sciences, Kermanshah, Iran; 2 Medical Biology Research Center, Kermanshah University of Medical Sciences, Kermanshah, Iran

**Keywords:** Hypoxia-inducible factor 1α, Leydig cells, Melatonin, Steroids, Testosterone

## Abstract

**Objective(s):**

This study examined the effects of melatonin treatment on steroidogenesis dysfunction and testosterone impairment, following CoCl_2_-induced hypoxia in TM3 Leydig cells.

**Materials and Methods:**

The TM3 cells were divided into four groups. The first group received no treatment. The MLT group was treated with a concentration of 1 mM melatonin. In the CoCl_2_ group, 0.2 mM CoCl_2_ was added to the medium to induce Hif1α overexpression. The MLT+CoCl_2_ group received 0.2 mM CoCl_2_ and 1 mM melatonin. After 24 hr treatment, the cells and supernatants were collected and used for further determination. The MTT assay was performed to estimate the decrease in cell viability throughout the CoCl_2_ and melatonin treatment. The mRNA and the protein levels were evaluated using Real-time PCR and Western blot analysis. The ELISA assay kit was used to detect the testosterone content.

**Results:**

CoCl_2_ treatment caused Hif1α overexpression in TM3 Leydig cells. Moreover, CoCl_2_ treatment of these cells led to considerable downregulation of Star, Hsd3b1, and Gata4 well as Mtnr1a and Mtnr1b mRNA/protein expression coupled with testosterone content repression in the cell culture medium. Melatonin administration in cells treated with CoCl_2_, decreased Hif1α mRNA/protein expression, but had no significant effect on Star, Hsd3b1, Gata4, Mtnr1a mRNA/protein expression, and the testosterone level in the cell culture medium. Melatonin caused recovery of decrease in the Mtnr1b gene and protein expression.

**Conclusion:**

There was no significant effect on steroidogenesis-related genes, proteins, and testosterone synthesis in the absence of gonadotropin treatment plus melatonin following CoCl_2_-induced hypoxia in TM3 Leydig cells.

## Introduction

It is estimated that infertility affects 8%-12% of couples of reproductive age globally (1). Reproductive disorders in men are responsible for 20% to 30% of infertility cases (2). Male fertility can be influenced by structural disorders, immunological defects, molecular and chromosomal abnormality, environmental and lifestyle factors, idiopathic factors, and endocrinological deficiency (3). Low O_2_ pressure (hypoxia) can cause steroidogenesis inhibition through enzymatic steroid production impairment (4, 5). Environmental exposure to cobalt chloride (CoCl_2_) can significantly affect male fertility in humans. Many animal models have confirmed the influence of acute and chronic exposure to CoCl_2_ on male reproduction (6, 7). CoCl_2_ is a hypoxia-mimicking agent *in vitro* and is a chemical inducer of hypoxia-inducible factor 1α (Hif1α), a subunit of Hif1(8). Hif1 consists of two subunits, Hif1α and Hif1β. Hif1α is rapidly hydroxylated and degraded in normoxia. Under hypoxic conditions, Hif1α stabilizes and, after its transfer from the cytoplasm to the nucleus and its association with Hif1β, the active Hif1 transcription complex is formed. This activated complex then attaches to the hypoxia response element in the regulatory regions of the target genes (9-12). It mediates the primary transcriptional responses to hypoxia and confers an adaptive role in proliferation, differentiation, energy metabolism, angiogenesis, metastasis, apoptosis, and steroidogenesis (13, 14). Studies have shown that steroidogenesis is affected by Hif1α protein, and disruption of Hif1α protein signaling can be linked to steroidogenesis disorders and reduced male reproductive function (5, 15). 

Testicular Leydig cells are located in the interstitial space of the seminiferous tubules in the testis. They are responsible for the synthesis and release of testosterone in mammals (16). The steroidogenic acute regulatory (Star) protein transports cholesterol from the outer mitochondrial membrane to the inner mitochondrial membrane. The enzyme Cyp11a1 converts cholesterol to pregnenolone in the inner membrane of the mitochondria. Pregnenolone is converted to testosterone through catalysis of the hydroxy-delta-5-steroid dehydrogenase, 3 beta- and steroid delta-isomerase 1 (Hsd3b1) enzyme in the smooth endoplasmic reticulum (17). 

Melatonin (N-acetyl-5-methoxytryptamine) is an indoleamine hormone composed of the amino acid tryptophan. It has been traditionally considered to be derived from the pineal gland and to interact with circadian rhythms (18-22). Sources of melatonin include the testes, retina, cornea, thymus, respiratory epithelium, cerebellum, bone marrow, gastrointestinal tract, and skin. Although melatonin can be found in many animal and plant foods, including fish, eggs, hazelnuts, coffee, and corn, melatonin supplements are usually made of synthetic melatonin (23). 

Recent evidence supports the role of melatonin in male reproduction. It plays a key role in mediating the influence of photoperiod on seasonal breeding in different animal species (24). Melatonin acts as an inhibitor of hypothalamic gonadotropin-releasing hormone (GnRH) production via the suprachiasmatic nucleus of the hypothalamus. GnRH influences the secretion of pituitary gonadotropin hormones, including follicle-stimulating hormone and luteinizing hormone (LH), which directly controls the function of Leydig cells. In addition, melatonin can cross the blood-testis barrier and enter Leydig cells. This hormone is also synthesized in the testis and interacts with its receptor on Leydig cells to confer a direct regulatory effect on the function of these cells independent of the hypothalamic-pituitary-gonadal axis. Melatonin affects male reproductive functions through a complex signaling pathway mediated by melatonin receptor 1a (Mtnr1a) and melatonin receptor 1b (Mtnr1b), that is consistent with cell-specific reproductive action (25, 26). Studies on rodents indicate that melatonin decreased the expression of Star and other key steroidogenic enzymes (27-31). Melatonin treatment has been shown to upregulate the expression levels of Star, Hsd3b1, and other key steroidogenic enzymes in the Leydig cells of mice fed a high-fat diet and H_2_O_2_-treated TM3 cells (32). Melatonin has also proved to modulate reproductive functions and upregulate the expression of the Star and GATA binding protein 4 (Gata4) transcription factors in seasonally breeding mammals (33). *In vitro* promotor analysis has revealed that the GATA4 protein is involved in the regulation of target genes in the Leydig cells. GATA4 is a novel downstream regulator of the cyclic adenosine monophosphate (cAMP)/protein kinase A (PKA) signaling pathway in steroidogenic cells. Further, GATA4 protein deficiency affects the steroidogenesis metabolic pathways. The StAR gene includes a regulatory sequence recognized by the GATA4 protein in the testes (16). Studies reported that long-term melatonin supplementation promotes StAR and GATA4 expression levels in goats and rams as well as enhances testosterone production, which resulted in improved sperm generation (34, 35). The current study used the TM3 mouse Leydig cell line model to investigate the antagonistic or synergistic effects of combining melatonin plus CoCl_2_ treatments on steroidogenesis-related genes and proteins expression as well as testosterone synthesis in TM3 Leydig cells.

## Materials and Methods

***Cell culture***

The TM3 cell line was purchased from the Pasteur Institute of Iran and was maintained in DMEM/F-12 medium (Bio-IDEA; Iran) supplemented with 10% fetal bovine serum (FBS; Gibco; USA) in an incubator at 37°C and 5% CO_2_. Cells attaining 70% to 80% confluency were divided into four groups in cell culture plates containing about 10^6^ cells in 3 ml of medium. The first group received no treatment. The MLT group was treated with a concentration of 1 mM melatonin (Sigma–Aldrich; USA). In the CoCl_2_ group, 0.2 mM CoCl_2_ (Merck; Germany) was added to the medium to induce Hif1α overexpression. The MLT+CoCl_2_ group received 0.2 mM CoCl_2_ and 1 mM melatonin. After 24 hr treatment, the cells and supernatants were collected and used for further determination.

***MTT assay***

About 5000 TM3 Leydig cells were seeded in each well of the 96-well plate. After 24 hr of treatment, a final concentration of 5 mg/ml of 3-(4, 5-dimethylthiazolyl-2)-2, 5 diphenyltetrazolium bromide (MTT; Sigma–Aldrich; USA) solution in phosphate-buffered saline (PBS) was added to each well. After incubation at 37 °C for 4 hr, the supernatant was removed and the formazan crystals produced in the cells were dissolved in 100 μl of dimethyl sulfoxide (DMSO; Merck; Germany), after which the cells were incubated with DMSO for 15 min. The absorbance of the plate was measured at a wavelength of 570 nm using a ELISA plate reader (Space fax 2100, Awareness, USA, reference 660 nm ). Each experiment was performed in triplicate.

***Real-time PCR analysis***

Purified total RNA (1 μg) was extracted using Trizol following the manufacturer’s protocol and was converted to cDNA using the Stem Gene cDNA synthesis kit (Stem Gene; Iran). Real-time PCR was performed using the SYBR Green qPCR master mix (Stem Gene; Iran) on a Runmei-q 200 system (Runmei; China). PCR primers (Table 1) were designed for the Mtnr1a, Mtnr1b, Gata4, Star, Hif1α, and Hsd3b1 genes. Gapdh was used as an internal control. The fold-change in mRNA was determined using the 2^−ΔΔCt^ method. Each experiment was performed in triplicate.

***Western blot analysis***

The total protein concentration was evaluated using the Bradford method. The protein was separated by 12% SDS-PAGE, then transferred to a nitrocellulose membrane. The membrane was blocked with 5% skim milk and incubated overnight at 4 °C with mild shaking using the anti-Hif1α (MAB1536, biotechne), anti-Hsd3b1 (ABIN2855488, antibodies), anti-Star (sc-166821, Santa Cruz), anti-Gata4 (ABIN6256227, antibodies), anti-Mtnr1a (ABIN361193, antibodies), anti-Mtnr1b (ABIN730318, antibodies), and anti-Gapdh (sc-365062, Santa Cruz) primary antibodies. The membrane was washed using PBS buffer, followed by incubation with the mouse anti-rabbit IgG-HRP (sc-2357, Santa Cruz) secondary antibody for 60 min at room temperature. The membrane was washed using PBS buffer. Immunocomplexes were detected using ECL Western blot detection reagents according to the manufacturer’s instructions in an X-ray processor (LD-14; China). The band density was determined using the JS2000 system (Bonnin Tech; China). Each experiment was performed in triplicate.

***Testosterone determination***

The cultured cells and supernatants were collected and an ELISA assay kit was used to detect the testosterone content according to the manufacturer’s instructions (AccuBind; USA). Each experiment was performed in triplicate.

***Statistical analysis***

Statistical analysis was performed in Graph Pad Prism 8 software (version 8.0; USA). Statistical significance was calculated using one-way ANOVA followed by Tukey and Dennett’s tests, being presented as mean ± Standard Deviation (mean ± S.D). Each experiment was performed in triplicate, with the sample size (n = 3) in each experiment, and *P*<0.05 was considered significant. The ranges of *P*-values were *P<0.05, **P< 0.01 and ***P<0.001.

## Results

***Cell viability of CoCl***_2_ ***and melatonin in mouse TM3 leydig cells***

**Figure 1:**
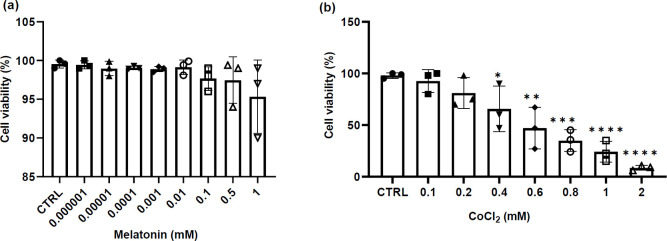
Cell viability evaluation performed using MTT assay. (a) TM3 cells treated with different concentrations of melatonin; (b) TM3 cells treated with different concentrations of CoCl_2_. The data are expressed as mean ± S.D (n=3, *P<0.05, **P<0.01, ***P<0.001 and ****P<0.0001 versus the CTRL group)

The MTT assay was performed to estimate the decrease in cell viability throughout the CoCl_2_ and melatonin treatment on TM3 Leydig cells. The cells were incubated with elevating melatonin concentrations (10^-6^ to 1 mM) for 24 hr. The results of the MTT assay indicated that the treatment of TM3 Leydig cells with different concentrations of melatonin did not decreased cell viability compared to the control group (Figure 1(a)); thus, the 1 mM melatonin concentration was selected for subsequent experiments. The cells were then incubated with elevating CoCl_2_ concentrations (0.1 to 2 mM) for 24 hr. It was determined that a 0.4 mM concentration of CoCl_2 _induced a significant decrease in cell viability compared to the control group (Figure 1(b)). The 0.2 mM concentration of CoCl_2_ was then selected for subsequent experiments.

***Upregulation of Hif1α by CoCl***_2_ ***reduced steroidogenesis-related genes and proteins levels*** ***in TM3 leydig cells***

**Figure 2:**
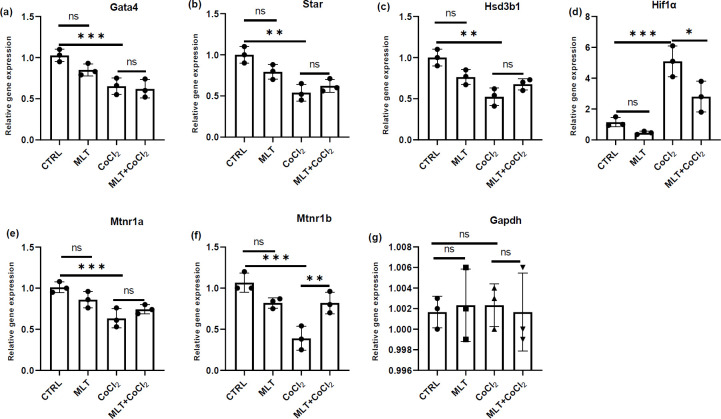
Real-Time PCR analysis of Gata4 (a), Star (b), Hsd3b1 (c), Hif1α (d), Mtnr1a (e), and Mtnr1b (f) gene expression where Gapdh was used as an internal control. The data are expressed as mean ± S.D (n=3, *P<0.05, **P<0.01 and ***P<0.001)

**Figure 3:**
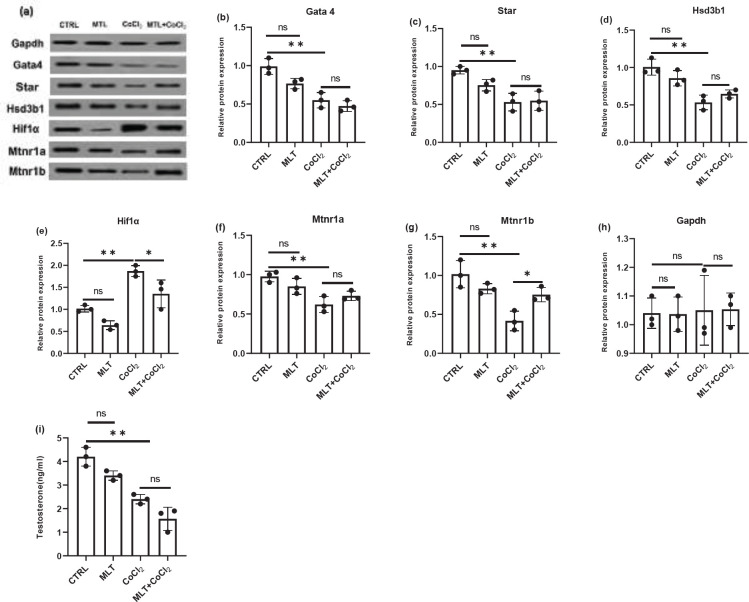
Western blot analysis of Gata 4 (a), (b); Star (a), (c); Hsd3b1 (a), (d); Hif1α (a), (e); Mtnr1a (a), (f); Mtnr1b (a), (g); protein expression where Gapdh was used as an internal control and testosterone concentration in the culture media (i). The data are expressed as mean ± S.D (n=3, *P<0.05, **P<0.01)

The mRNA and protein expression of Hif1α was upregulated after CoCl_2_ treatment compared to the control group in TM3 Leydig cells (Figures 2(d), 3(a), and 3(e)). The protein and mRNA levels of Gata4, Star, and Hsd3b1 decreased significantly compared to the control group after CoCl_2_ treatment (Figures 2(a-c), 3(a-d)). Melatonin treatment showed no significant effect on the expression of Hif1α, Gata4, Star, and Hsd3b1 genes compared to the control group (Figure 2(a-d)). Similar results were obtained in the Western blot test results for the Hif1α, Gata4, Star, and Hsd3b1 protein levels (Figures 3(a-e)). Melatonin treatment caused recovery of Hif1α mRNA and protein expression accumulation following CoCl_2_ treatment (Figures 2(d), 3(a), and 3(e)). The melatonin treatment revealed no significant effect on the downregulation of Star, Gata4, and Hsd3b1 mRNA and protein expression following CoCl_2_ treatment (Figures 2(a-c), 3(a-d)).

***CoCl***_2_ ***decreased*** ***testosterone production in TM3 leydig cells***

CoCl_2_ treatment significantly decreased the testosterone content in the culture medium compared to the control group. In addition, melatonin showed no significant effect on the testosterone content following the CoCl_2_ treatment (Figure 3(i)). 

***Decrease of melatonin receptor expression in CoCl***_2_ ***-treated mouse TM3 Leydig cells recovered by melatonin***

Real-time PCR and Western blot analysis revealed that membrane receptors Mtnr1a and Mtnr1b were expressed excessively in the control group. CoCl_2_ treatment decreased the mRNA and protein expression of Mtnr1b compared to the control group (Figures. 2(e), 2(f), 3(a), 3(f), 3(g)). Melatonin treatment caused recovery of Mtnr1b mRNA and protein expression deficiencies of the CoCl_2_-treated cells (Figs. 2(f)*, *3(a), 3(g)).  

**Table 1:** Primers used in this study for Real-time PCR analysis

Gene	Primer sequence	Size (bp)	GeneBank ACC
***Gapdh*** **-forward**	CATCACTGCCACCCAGAAGACTG	152	NM-001289726.1
***Gapdh*** **-reverse**	ATGCCAGTGAGCTTCCCGTTCAG		
***Gata4*** **-forward**	GCCTCTATCACAAGATGAACGGC	148	NM-001310610.1
***Gata4*** **-reverse**	TACAGGCTCACCCTCGGCATTA		
***Mtnr1a*** **-forward**	AACCTGCTGGTCATCCTGTCTG	113	NM-008639.3
***Mtnr1a*** **-reverse**	GGGATAAGGGTAAACAGCCACC		
***Mtnr1b*** **-forward**	TCTCAGTGCTCAGGAACCGCAA	136	AB377276.1
***Mtnr1b*** **-reverse**	AAGGACCCAACCGTCACGGATA		
***Hsd3b1*** **-forward**	AGAACTGCAGGAGGTCAGAGCT	117	NM-008293.4
***Hsd3b1*** **-reverse**	GGCATCCAGAATGTCTCCTTCC		
***Star*** **-forward**	GTGCTTCATCCACTGGCTGGAA	112	NM-011485.5
***Star*** **-reverse**	GTCTGCGATAGGACCTGGTTGA		
***Hif1α*** **-forward**	CCTGCACTGAATCAAGAGGTTGC	109	NM-001313919.1
***Hif1α*** **-reverse**	CCATCAGAAGGACTTGCTGGCT		

## Discussion

The results of this investigation confirmed a correlation between CoCl_2_-induced hypoxia and Hif1α accumulation with steroidogenesis-related genes and proteins downregulation as well as testosterone deficiency in TM3 Leydig cells. These results are in agreement with those of previous reports where Hif1α protein was involved in the regulation of Hsd3b1 and Star protein expression plus testosterone production in mouse TM3 Leydig cell line (15). It has been documented that overexpression of HIF1α protein in granulosa cells and canine lutein cells treated with CoCl_2_ exhibited similar effects on steroid synthesis (14, 36). Other signaling proteins and nuclear transcription factors also are involved in cell cycle arrest following HIF1α protein overexpression (14). Downregulation of basal and cAMP-induced Star protein expression as well as steroidogenesis mediated by Hif1α protein in murine granulosa cells under hypoxia has been demonstrated (37). Recent evidence has suggested that Hif1α protein stabilization under severe hypoxia produces detrimental effects on steroidogenesis. Possible regulatory mechanisms for the Hif1α protein involve cAMP production, regulating PKA activity, and phosphorylation of target transcription factors (38). It has been reported that HIF1α protein stabilization in a hypoxic environment may contribute to high intracellular reactive oxygen species (ROS) levels in Leydig cells. ROS is known to impair steroidogenesis by inducing oxidative stress, which leads to a reduction in the Bcl2/Bax ratio, p53 gene upregulation, and downregulation of the Bcl2 gene. Alterations in the ratio of Bcl2/Bax have been shown to lead to cytochrome C release, which promotes caspase-9 and activates the caspase-3 signaling cascades, leading to apoptosis (39). Further, the Hif1α protein is capable of regulating the transcription of the mouse Hsd3b1 gene by influencing its promotor activity (40). 

Melatonin is a neuroendocrine molecule that modulates endogenous patterns with photoperiod changes (26). Melatonin signaling pathways in target cells lead to activation of MTNR1A and MTNR1B, resulting in inhibition of cAMP activity through coupling with Gi protein. Inhibition of cAMP can regulate PKA activity and reduce phosphorylation of cAMP response element-binding protein, its downstream effector, to decrease the expression of genes required for steroidogenesis, including StAR, HSD3B1, and GATA4 (29, 30, 35). 

The present study revealed that melatonin treatment had no significant effect on the expression of Gata4, Star, and Hsd3b1 genes and proteins in TM3 Leydig cells compared to the control group. In addition, the testosterone concentration in the cell culture medium remained unchanged in these cells. These observations are in contrast with evidence that MA-10 Leydig cell line treatment with human chorionic gonadotropin (hCG)/cAMP analogue for stimulation of testosterone synthesis alone or with the hCG/cAMP analogue plus melatonin at different dosages resulted in downregulation of the Star protein expression and steroidogenesis. Melatonin attenuated Star protein expression and testosterone synthesis was cAMP pathway-dependent and stimulated by the hCG or cAMP analogue (28). This contrast may be due to the absence of gonadotropin treatment plus melatonin. The mechanism of testosterone production is regulated by multiple factors, where hCG or LH is widely used for the stimulation of testosterone production in cells (17). Another study has reported that melatonin treatment caused a significant decline in the expression of Gata4 and SF-1, crucial steroidogenic enzymes, and testosterone production in the TM3 Leydig cell line under LH treatment (29). The results of the present study demonstrated that melatonin suppressed the adverse effects of CoCl_2_ treatment in Hif1α mRNA and protein expression. Recent evidence has demonstrated that melatonin exerts its effect on metabolic pathways and cancer treatment by reducing the HIF1α protein (41). Administration of melatonin has been shown to decrease the HIF1α protein level inside the tumor mass and prevented the growth of tumors in mice (42). In another study, HIF1α protein was decreased in HCT116 cells using melatonin under hypoxia. HIF1α protein stabilization under hypoxic conditions has been shown to cause ROS production. The anti-oxidant properties of melatonin have been shown to remove intracellular ROS and destabilize the HIF1α protein (43). 

The data revealed that mRNA and protein expression of Mtnr1b decreased following CoCl_2_**-**induced hypoxia in TM3 Leydig cells and that melatonin treatment caused recovery of the Mtnr1b gene and protein deficiencies. Similar to the present study, it has been reported that the Mtnr1a gene and protein deficiencies in hypoxic-ischemic mice with brain injuries were reduced by applying melatonin *in vivo*. Melatonin receptor antagonist was used to demonstrate the neuroprotective effect of melatonin in neonatal hypoxic-ischemic mice with brain injuries (44). Another study speculated that melatonin exerts its protective effect in hypoxia-induced retinopathy through preserving melatonin receptors (45). 

## Conclusion

In summary, the current study results indicated that CoCl_2_ reduced TM3 cell line steroidogenesis-related genes, proteins, and testosterone synthesis. Melatonin destabilized the Hif1α gene and protein as well as caused recovery of the Mtnr1b gene and protein deficiencies following CoCl_2_ treatment. There was no significant effect on steroidogenesis-related genes, proteins, and testosterone synthesis in the absence of gonadotropin treatment plus melatonin. 

## Authors’ Contributions

S K and M G designed the experiments; S K performed experiments and collected the data; S K and M G discussed the results and strategy; M G supervised, directed, and managed the study; S K, C J, KM, F B, and M G approved the final version to be published. 

## Conflicts of Interest

The authors declare no competing interests.
